# Performance Analysis of Beidou-2/Beidou-3e Combined Solution with Emphasis on Precise Orbit Determination and Precise Point Positioning

**DOI:** 10.3390/s18010135

**Published:** 2018-01-05

**Authors:** Xiaolong Xu, Min Li, Wenwen Li, Jingnan Liu

**Affiliations:** 1 GNSS Research Center, Wuhan University, 129 Luoyu Road, Wuhan 430079, China; xlxu@whu.edu.cn (X.X.); cheeselee@whu.edu.cn (W.L.); 2 Collaborative Innovation Center of Geospatial Technology, 129 Luoyu Road, Wuhan 430079, China

**Keywords:** BeiDou-3e, code bias, precise orbit determination, ambiguity fixing, precise point positioning

## Abstract

In 2015, the plan for global coverage by the Chinese BeiDou Navigation Satellite System was launched. Five global BeiDou experimental satellites (BeiDou-3e) are in orbit for testing. To analyze the performances of precise orbit determination (POD) and precise point positioning (PPP) of onboard BeiDou satellites, about two months of data from 24 tracking stations were used. According to quality analysis of BeiDou-2/BeiDou-3e data, there is no satellite-induced code bias in BeiDou-3e satellites, which has been found in BeiDou-2 satellites. This phenomenon indicates that the quality issues of pseudorange data in BeiDou satellites have been solved well. POD results indicate that the BeiDou-3e orbit precision is comparable to that of BeiDou-2 satellites. The ambiguity fixed solution improved the orbit consistency of inclined geosynchronous orbit satellites in along-track and cross-track directions, but had little effect in the radial direction. Satellite laser ranging of BeiDou-3e medium Earth orbit satellites (MEOs) achieved a standard deviation of about 4 cm. Differences in clock offset series after the removal of reference clock in overlapping arcs were used to assess clock quality, and standard deviation of clock offset could reach 0.18 ns on average, which was in agreement with the orbit precision. For static PPP, when BeiDou-3e satellites were included, the positioning performance for horizontal components was improved slightly. For kinematic PPP, when global positioning satellites (GPS) were combined with BeiDou-2 and BeiDou-3e satellites, the convergence time was 13.5 min with a precision of 2–3 cm for horizontal components, and 3–4 cm for the vertical component.

## 1. Introduction

According to the experience of the first generation of the Chinese BeiDou Navigation Satellite System, the development of the second generation has been separated into two phases: (1) service for the Asia-Pacific region by the end of 2012 (BeiDou-2) and (2) global coverage by the end of 2020 (BeiDou-3) [1,2]. The BeiDou-2 constellation that provides the regional service consists of three types of satellites: geostationary orbit (GEO), inclined geosynchronous orbit (IGSO), and medium Earth orbit (MEO). The constellation for regional service consists of 14 satellites, including five GEOs (pseudo random noise (PRN) C01–C05), five IGSOs (PRN C06–C10), and four MEOs (PRN C11–C14) [3]. Because the M05 satellite (PRN C13) had already stopped transmitting signals since 21 October 2014, a new IGSO (I06) was launched to supply regional coverage and has been in service since March 2016.

With the development of BeiDou-2, many studies have been carried out on various aspects of this system. In comparison with the Global Positioning System (GPS) and the GLObal Navigation Satellite System (GLONASS), BeiDou-2 satellite pseudorange observations show obvious systematic biases in different frequencies for different constellations, which were first mentioned by Hauschild et al. [4] and Shi et al. [5], and then were subsequently diagnosed and modeled using piece-wise linear functions with respect to satellite elevations by Wanninger [6]. As for the BeiDou precise orbit determination (POD) research, six MGEX (Multi-GNSS Experiment) analysis centers (ACs) have been routinely providing GNSS precise orbit and clock products since 2012 on the basis of MGEX stations, namely, the Center for Orbit Determination in Europe (CODE), GeoForschungsZentrum Potsdam (GFZ), Centre National d’Etudes Spatiales / Collecte de Localisation Satellite (CNES/CLS), the Japan Aerospace Exploration Agency (JAXA), Technische Universität München (TUM), and Wuhan University (WHU). Guo et al. [7] assessed BeiDou orbits provided by MGEX ACs, which showed that the precision of day boundary discontinuities for all AC products were on the decimeter to meter scale for GEOs, 1–3 dm for IGSOs, and 10–27 cm for MEOs. Moreover, the performance of precise point positioning (PPP) by the BeiDou-2 system has also been assessed. The horizontal and vertical positioning accuracies were found to be better than 1 cm and 3 cm in static mode, and better than 2–3 cm and 10 cm in kinematic mode. The PPP convergence time for the BeiDou-2 system was only about 50 min [8,9,10].

To implement the plan for global service, five next-generation experimental satellites (two IGSOs and three MEOs), named BeiDou-3e [11], have been launched since 2015 to test satellite payloads and global service signals. Inter-satellite link (ISL) payloads have been assembled on the experimental satellites, and observational data of ISL has been obtained and processed for autonomous orbit determination [12]. The BeiDou-3e satellites transmit signals at five frequencies, namely, B1I and B3I legacy signals, similar to those from BeiDou-2 satellites, as well as modernized signals in the B1C, B2a, and B2b bands. Furthermore, Zhang et al. [11] investigated the consistency of the carrier phase for BeiDou-3e satellites using the differential ionosphere-free combination with respect to the triple-frequency carrier phase using ionosphere-free and geometry-free combination. No significant bias variations were observed for any of the next-generation BeiDou-3e satellites, although these have been found in BeiDou-2 and GPS Block IIF satellites signals owing to unstable inter-frequency clock offsets. Tan et al. [13] calculated the BeiDou-3e orbit and assessed its precision by overlapping comparison and satellite laser ranging (SLR) based on nine BeiDou-3e stations and 52 BeiDou-2 stations, and the precisions of the radial components for BeiDou-3e IGSOs and MEOs were 10 cm and 40 cm, respectively. Xie et al. [14] obtained initial results for the precise orbits of BeiDou-3e satellites by the real-valued ambiguity solution based on 11 BeiDou-3e stations and 34 BeiDou-2 stations, and the precision achieved the levels of about 20–60 cm in terms of three-dimensional root mean square (RMS) values. 

With the addition of the five BeiDou-3e satellites, the number of operational BeiDou satellites in orbit has reached 19, which has further improved the spatial geometry in the service area. Thus, it is interesting to analyze the combined solution for POD and PPP of all available BeiDou satellites in service, including BeiDou-2 and BeiDou-3e satellites. The availability and collection of data are described in Section 2. In Section 3, the signal performance is analyzed in details. Section 4 describes the POD strategy, and then the results are analyzed and validated. The PPP results are presented in Section 5. Conclusions and further work are discussed in the final section.

## 2. Data Availability and Collection

The current BeiDou-2 satellites transmit signals at three frequencies, namely, B1(1561.098 MHz), B2 (1207.140 MHz), and B3 (1269.520 MHz), on channel I and channel Q. Channel I is used for civil services and channel Q is used for military purposes [3]. As part of the development of the plan for global coverage and for testing the new BeiDou signal components, the new BeiDou-3e satellites retain the original B1I and B3I signals, but the B2I signal has been replaced by three new experimental signals, which are named B1C (1575.42 MHz), B2a (1176.45 MHz), and B2b (1207.17 MHz) [15,16]. The legacy BeiDou B1, B2 and B3 frequency signal structures were firstly published in the BeiDou Signal In Space Interface Control Document in December, 2012; however, the new signal specifications are still in the phase and kept confidential. Thus, commercial receivers cannot decode the new B2 codes currently.

**Figure 1:**
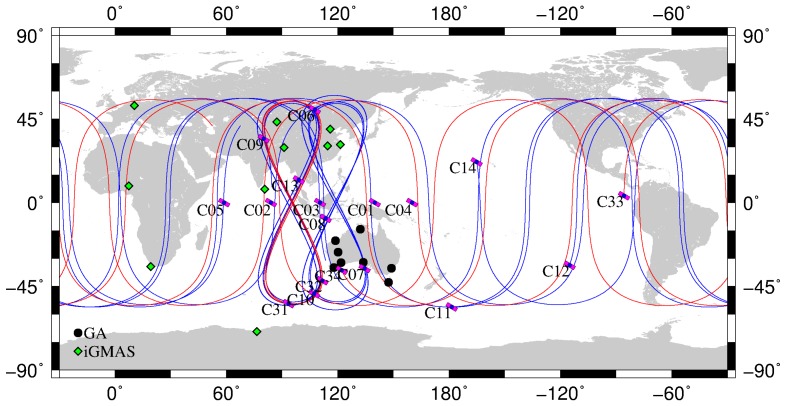
Stations used for precise orbit determination (POD) of BeiDou satellites. The green diamonds represent iGMAS stations and the black circles represent GA tracking stations. The blue and red lines indicate the trajectories of the BeiDou-2 and BeiDou-3e satellites, respectively.

With the assistance of the International GNSS Service (IGS) Multi-GNSS Experiment (MGEX) plan [17,18], about 209 multi-GNSS stations have been set up in the IGS network, of which 161 stations are designed for tracking BeiDou-2 satellites (up to 21 August 2017). However, most receivers in the MGEX network cannot track the BeiDou-3e B1I and B3I signals, which is mainly due to old firmware versions. In order to monitor BeiDou satellite signal precision and to improve BeiDou service and performance, the international GNSS Monitoring and Assessment Service (iGMAS) was initiated and developed by China through deploying a worldwide multi-GNSS monitoring stations, which enables global tracking of BeiDou satellites [19]. Currently, 10 of the iGMAS stations are equipped with receivers manufactured by the 20th and 54th Institutes of the China Electronics Technology Group Corporation, and can track BeiDou-3e signals. In addition, 14 stations, from Geoscience Australia (GA) network, are able to track the original BeiDou-3e B1I and B3I signals. Table 1 lists information on these capable receivers, and their distribution is shown in Figure 1. The iGMAS and GA data from 21 January 2017 (day of year (DOY) 021) to 19 March 2017 (DOY 079) have been collected and subsequently used for analyses in this study. The selected length of data period is proved to be effective for evaluation of orbit precision under the same status of satellites and distribution of stations [14,20].

## 3. Signal Performance

**Figure 2:**
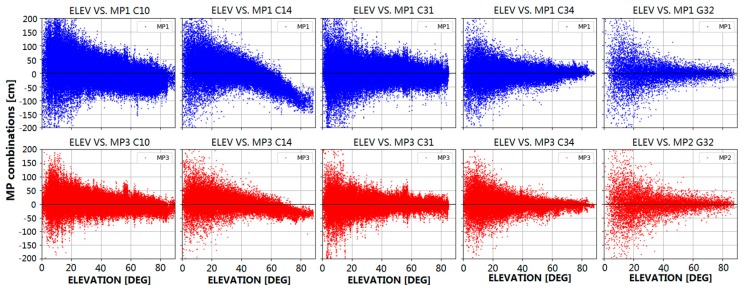
Multipath (MP) combinations for BeiDou-2 (C10, C14), BeiDou-3e (C31, C34), and GPS (G32) satellites with respect to elevation. For BeiDou satellites, MP1 and MP3 represent the B1 and B3 frequency MPs, respectively, whereas for global positioning satellites (GPS) (right column), MP1 and MP2 represent the L1 and L2 frequency MPs. Blue dots represent MP1 and red dots represent MP3/MP2.

The pseudorange code bias found in BeiDou-2 signals has already been verified, analyzed, and corrected [4,6,21,22]. The bias has been shown to be most probably caused by satellite components other than the receiver surroundings. Variations in the code observations were first detected using the multipath (MP) combination. This combination, which is both geometry-free and ionosphere-free, was presented by Estey and Meertens [23] and applied in the TEQC tool. The combination is defined as:

(1) $$MP_{i}=P_{i}-\frac{f_{i}^{2}+f_{j}^{2}}{f_{i}^{2}-f_{j}^{2}}\lambda_{i}\varphi_{i}+\frac{2f_{j}^{2}}{f_{i}^{2}-f_{j}^{2}}\lambda_{j}\varphi_{j}-B_{i},i\neq j$$

where *f* is frequency, P and φ represent the code range and carrier phase observables, respectively, and λ represents wavelength of carrier phase, whereas the subscripts *i* and *j* are used to denote different number of frequencies. The bias term $B_{i}$ mainly contains the linear combination of the phase ambiguities and the constant part of the hardware delays. In general, the carrier phase MP is much lower than the pseudorange MP and can be neglected in the MP combination. If no cycle slip occurs during successive epochs, the bias $B_{i}$ is assumed to be constant and should be replaced by the average MP. As GEOs remain nearly stationary relative to ground stations, and the surroundings of the stations also remain the same, the relation between elevation angle and MP is not analyzed in this paper. In addition, as only IGSOs and MEOs are presented in the BeiDou-3e constellation, we demonstrate the B1 frequency MP (MP1) and B3 frequency MP (MP3) characteristics for representative BeiDou-2 and BeiDou-3e satellites using the B1I and B3I signals of IGSOs and MEOs. The behavior of MP combinations for BeiDou and GPS satellites versus elevation is shown in Figure 2. In this paper, BeiDou-2 IGSO (PRN C10) and MEO (PRN C14), BeiDou-3e IGSO (PRN C31) and MEO (PRN C34), and GPS Block IIF (PRN G32) are selected as representative examples.

The data shown in Figure 2 were collected from all the stations distributed in Asia-Pacific region, which is indicated in Figure 1. The blue and red dots correspond to the B1I and B3I frequencies, respectively. As mentioned in [6], the MP bias is satellite-induced and linearly dependent on the elevation of the satellite. As seen in Figure 2, the MP values for the BeiDou-2 satellites are not uniformly distributed near zero but display an obvious tendency with elevation from positive values (low elevation) to negative values (high elevation), in which the slope for the B1I signal is greater than that for the B3I signal, and the slope for MEOs is greater than that for IGSOs. This phenomenon is related to the BeiDou satellite types and can be clustered into IGSO and MEO groups, and the maximum bias in the B1I signal for MEOs is over 1 m, close to the zenith. For BeiDou-3e satellites, no obvious variations in signals as a function of elevation can be found. Obviously, the satellite-induced MP bias is almost eliminated, and the MP values for BeiDou-3e satellites fluctuate slightly to the same extent as those for GPS satellites. After detrending the MP series, the fluctuation in MP values for IGSOs is larger than that for MEOs. This may be related to the satellite altitude: IGSOs are in geosynchronous orbit at an altitude of 36,000 km, which is much higher than the orbit of MEOs (22,000 km), and the carrier-to-noise density ratio for IGSOs is lower than that for MEOs. Moreover, the MP for the B3I signal is smaller than that for the B1I signal and the noise in the B3I signal is also smaller than that in the B1I signal, which may be related to the precision of the code element. 

**Figure 3:**
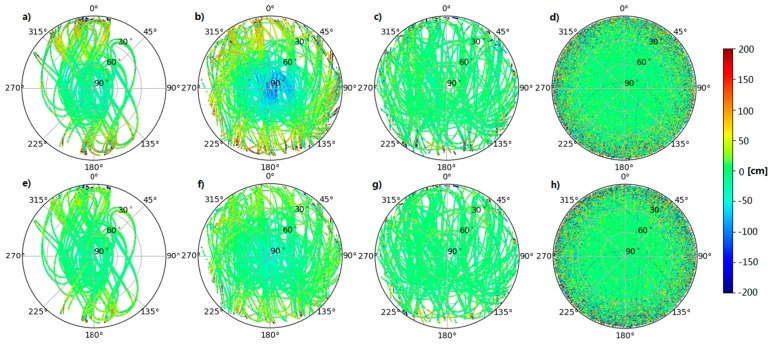
MP combinations for BeiDou-2 and BeiDou-3e satellites as a function of azimuth and elevation. The first and second columns represent BeiDou-2 inclined geosynchronous orbits (IGSOs) and medium Earth orbits (MEOs), respectively, the third column represents BeiDou-3e satellites, and the last column represents GPS satellites. The MP for the B1I and L1 signals is shown at the top and the MP for the B3I and L2 signals is shown at the bottom.

Figure 3 shows scatter plots of MP for BeiDou-2 IGSO and MEO, BeiDou-3e, and GPS satellites as a function of the azimuth and elevation from the aforementioned tracking stations, where Figure 3a–c represent the B1I pseudorange, Figure 3d represents the GPS L1 signal, and Figure 3e–g represent the B3I pseudorange, and (h) represents the GPS L2 signal. It can be observed that the MP is independent of the azimuth for both BeiDou-2 and BeiDou-3e satellites and is also independent of the elevation for BeiDou-3e satellites, in the same way as for GPS satellites.

The average and RMS values of the MP combinations are listed in Table 2. The data used in Table 2 include values for elevation angles ranging from 0 to 90°. As seen in Table 2, there is an average bias of more than 1 cm for the B1I and B3I signals in BeiDou-2 IGSOs and MEOs, but this bias is absent for BeiDou-3e and GPS satellites. In addition, the RMS values for BeiDou-3e satellites are on the same level as those for GPS satellites, which represents a remarkable improvement in comparison with BeiDou-2 satellites.

## 4. Precise Orbit Determination (POD)

### 4.1. POD Strategy

In order to analyze the POD performance for BeiDou-3e satellites, only stations with the ability to capture BeiDou-3e and BeiDou-2 signals were used. Position And Navigation Data Analyst software (PANDA), which was developed by the GNSS Research Center at Wuhan University [24], was used for POD in this study. In the combined POD of BeiDou-3e and BeiDou-2 satellites, the prior position and velocity of the satellites were imported from the broadcast ephemeris provided by iGMAS stations. The orbit parameters included the initial position and velocity, five parameters of solar radiation pressure (SRP) in the Empirical CODE Orbit Model (ECOM) [25], with a constant acceleration bias in along-track direction [26]. The entire data processing workflow for the combined POD of BeiDou-2 and BeiDou-3e satellites can be separated into two steps. Firstly, GPS precise orbit and 30 s interval clock products provided by IGS were used to estimate the coordinates, zenith troposphere delays (ZTDs), and receiver clock offsets of the stations. Secondly, the parameters estimated above were fixed and the BeiDou orbit parameters, together with the clock offsets of the satellites, were estimated in batch mode. 

**Figure 4:**
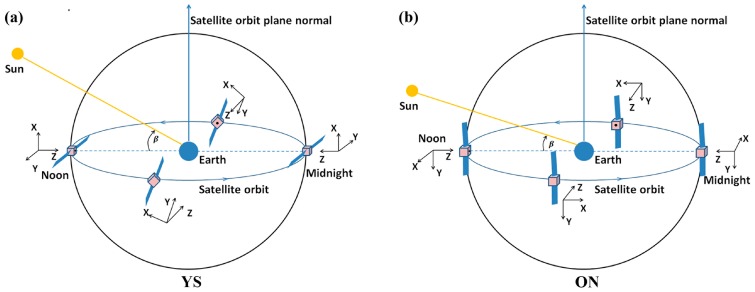
BeiDou-2 satellite attitude control modes: yaw-steering (YS) (**a**) and orbit-normal (ON) (**b**). In YS mode, the satellite rotates around the z axis, and the x axis remains yawing all the time, while during the ON period, the x axis stops yawing and is consistent with the velocity throughout.

Satellite attitude is essential for solar radiation calculations as well as for phase center offset (PCO) corrections. In general, in order to transmit navigational signals to the ground and receive energy from the Sun, satellites must be ensured that (1) their transmitting antenna points to the Earth and (2) their solar panels are perpendicular to incoming sunlight [27]. Two control modes, namely, yaw-steering (YS) and orbit-normal (ON), are used in BeiDou satellites system to simplify attitude control during eclipse periods [28,29,30]. Generally, in the satellite-fixed reference frame, center of satellite mass is set as the coordinate origin, the z axis is along the antenna transmitting direction and points to the geocenter, the y axis is along the solar panels and is perpendicular to either the satellite-Sun direction (YS mode) or the satellite orbital plane (ON mode), and the x axis completes the right-handed coordinate system. As shown in Figure 4, in YS mode, a satellite always conforms to two laws of satellites attitude control (mentioned above) and the satellite bus constantly yaws, whereas in ON mode the GNSS antenna points to the Earth but the satellite bus does not yaw and the solar panels are perpendicular to the orbital plane. Of the BeiDou-2 satellites, GEOs are constantly operated in ON mode, while IGSOs and MEOs are operated in both YS and ON modes. YS mode is used when the absolute value of the solar elevation angle (beta angle) above the orbital plane is greater than 4°; otherwise, ON mode is utilized [29]. 

According to an analysis by Guo et al. [28], orbit precision declines dramatically during ON periods, and therefore a constant force in along-track is introduced to compensate for the ECOM output. The orbit precision during ON period is improved to the same level as that during YS period, but it is still low during transitions between attitude control modes. In this study, the five parameters in ECOM together with an additional acceleration bias were used for the combined POD of BeiDou-2 and BeiDou-3e satellites. It should be noted that YS mode alone, as in the case of GPS satellites, was used for BeiDou-3e satellites according to information provided by Operational Control Center (OCC). 

To further improve the precision of POD, integer ambiguity resolution (AR) can be crucial. The method of ambiguity fixing used in this study was proposed by Ge et al. [31]. The ambiguities of the undifferenced ionosphere-free combination were divided into wide-lane (WL) and narrow-lane (NL) ambiguities. WL and NL ambiguities are linear combinations of phase ambiguities and can be expressed as:

(2) $$\{\begin{array}{c} N_{WL}=N_{i}-N_{j}\\ N_{NL}=N_{i}+N_{j} \end{array}$$

where N represents carrier phase ambiguity, and the subscripts *i* and *j* are used to denote different number of frequencies. The geometry-free WL phase ambiguity was initially constrained to integer values through the Hatch-Melbourne-Wübbena (HMW) combination [32,33,34], and then an attempt was made to fix the geometry-based NL phase ambiguity. In addition, in our experiments the GEO ambiguities were not fixed due to their poor geometry. When implementing AR during a POD process, the pseudorange measurement quality can have significant impacts on the resolution success rate of the geometry-free WL phase ambiguities [35]. The code bias correction model supplied by Wanninger and Beer was adopted to eliminate pseudorange errors in the BeiDou-2 IGSOs and MEOs signals [6]. With respect to the BeiDou-3e signal performance, there was no obvious code bias and corrections were not needed.

Table 3 summarizes the observational models and force models used in the POD processing. Specifically, the station coordinates were calculated as constants and the ZTDs were estimated as piece-wise constants every 120 min, whereas the clock offsets of the satellites and stations were estimated on an epoch basis and constrained by a random walk model. Owing to the different frequencies and signal modulation methods used by GPS and BeiDou, there is a bias in receiver clock offsets between these two systems relative to the reference time. This bias is stable and can be estimated as inter-system bias [36]. In addition, as the tracking stations used in our analysis were regional stations, the Earth rotation parameters were not estimated but were fixed in accordance with the international reference time series for the Earth orientation parameters, referred as “IERS C04” (Combined 04), which was provided by the Earth Orientation Center of the International Earth Rotation Service. For the BeiDou-2 satellites, only the initial PCO values were supplied by the IGS-MGEX without phase center variation (PCV) parameters. Hence, we used the PCO and PCV values calculated by Wuhan University to correct data for the IGSOs and MEOs [7]. Unfortunately, no precise PCO parameters for C13 are available. There is only the initial value of 60, 0, and 110 cm in satellite-fixed coordinates for C13. For BeiDou-3e satellites, PCO values are provided by OCC with no PCV information, as listed in Table 4.

### 4.2. Orbit Validation

#### 4.2.1. Orbit Overlap Comparisons

Orbit overlap comparisons are widely used to validate the internal consistency of orbits, which can evaluate precision at each epoch in overlapped time of the orbit. Thus, the method based on the overlap between two adjacent arcs is utilized in this study. We compared the satellite positions on the middle day of the first three-day arc and the first day of the second arc.

As mentioned above, data from DOY 021 to DOY 079 in 2017 were considered in this study. Table 5 shows the RMS results of an overlap comparison of the real-valued ambiguity solution. As listed in Table 5, the results of the orbit comparison of BeiDou-2 and BeiDou-3e IGSOs and MEOs were on the same scale: the radial component was about 4.3–6.5 cm, the cross-track component was about 11.5–19.4 cm, and the along-track component, which was the largest, was approximately 13.4–21.5 cm. The three-dimensional RMS value was about 19.7–27.4 cm. Owing to poor geometry, the RMS value of the along-track component was about 46.6–73.4 cm for GEOs, which was much larger than those for IGSOs and MEOs, but the cross-track component was about 3.2–8.0 cm, which was much smaller than those for IGSOs and MEOs. The radial component was about 6.0 cm and was thus on the same scale as those for IGSOs and MEOs.

**Figure 5:**
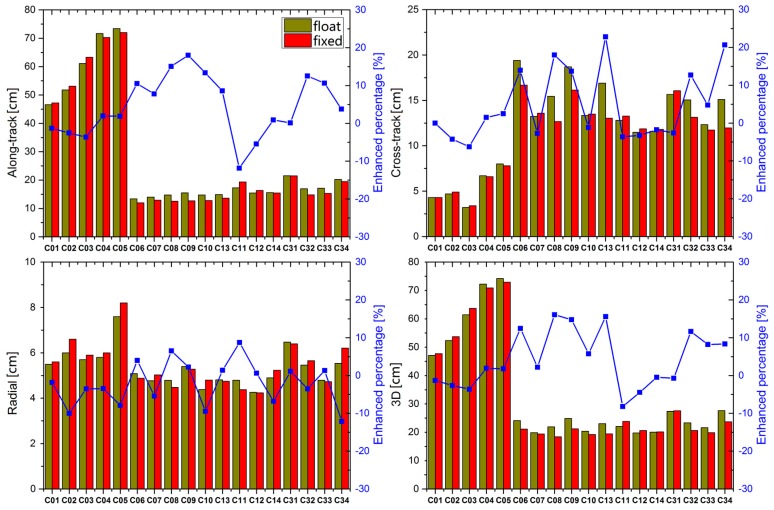
Comparison of RMS values of orbit overlap before and after ambiguity fixing. The dark yellow and red bars represent real-valued ambiguity (float) and ambiguity fixed (fixed) solutions, respectively.

In this study, the ambiguity was not fixed for GEOs owing to their poor geometry, but the performance of IGSOs and MEOs was still inspiring, and our results showed that the ambiguity fixing rate for IGSOs and MEOs was about 80%. The comparative results for BeiDou-2 and BeiDou-3e satellites before and after successful fixing are shown in Figure 5. As depicted in Figure 5, all the radial components were enhanced very slightly, but the along-track and cross-track components were enhanced by almost 10% for IGSOs. The performance of MEOs improved to a less extent than that of IGSOs, and some satellites were even worse than that of real-valued ambiguity solution, which may be due to the large tracking gap occurred in MEOs when they were outside the Asia-Pacific zone, as shown by the locations of the tracking stations in Figure 1. It should be noteworthy that continuous observation can be achieved only for IGSOs, but not for MEOs, which results in lower precision of parameters for MEO, compared with that for IGSO. Owing to discontinuous observation for MEOs, poor orbit precision of MEOs was displayed. All orbit-related parameters were solved in batch mode, and ambiguity fixing for such a short period had little effect on the results for the entire arc, and it was not long enough to improve the orbit precision of MEOs significantly. The performance of BeiDou-2 GEOs was almost unaffected because the ambiguity remained real-valued.

#### 4.2.2. SLR Validation

SLR is an independent optical system and is treated as an external validation method, in contrast to the GNSS L band. Errors related to the propagation path can be eliminated accurately with a ranging accuracy of less than 1 cm, which benefits from the high signal frequency. As the optical system is located on the ground surface, values measured by SLR are mainly assigned to the radial component of the orbit. The precise coordinates and velocities of the tracking stations are provided by International Laser Ranging Service (ILRS) [41]. SLR observations can be used to check the geometric distances from satellites to SLR tracking stations. SLR supplies effective methods for diagnosing defects in SRP models [42], or for combined orbit determination of GNSS [43] and low Earth orbit satellites [44].

All the BeiDou-2 satellites are equipped with a laser retroreflector array (LRA), but only C01, C08, C10, C11, and C13 among the BeiDou-2 satellites and all the BeiDou-3e satellites are tracked by ILRS, where C31 has not been tracked by ILRS since 18 December 2016. The LRA offsets supplied by the OCC are listed in Table 6.

Amongst all the SLR tracking stations, during test data period in our analysis, from DOY 021 to DOY 079 in 2017, 20 stations have been tracking the BeiDou-2 satellites, whereas only nine stations have been tracking the BeiDou-3e satellites. Thus, the number of normal points (NPs) for BeiDou-3e satellites is much fewer than that for BeiDou-2 satellites: three SLR stations tracked C32 (Yarragadee: 65 NPs, Shanghai: 10 NPs, Changchun: 10 NPs); four stations for C33 (Yarragadee: 13 NPs, Matera: 8 NPs, Monument Peak: 6 NPs, Herstmonceux: 6 NPs); and nine stations for C34 (Yarragadee: 15 NPs, Shanghai: 9 NPs, Changchun: 4 NPs, Matera: 6 NPs, Monument Peak: 7 NPs, Herstmonceux: 7 NPs, Tahiti: 5 NPs, Beijing: 3 NPs, Graz: 2 NPs). However, orbit precision can still be validated approximately by time series and statistical information of SLR. The results of SLR validation are listed in Table 7. It is noted that some abnormal data have been removed from the raw dataset, but the data utilization rates were all greater than 95%. The RMS values of the SLR validation results are also shown in Table 7. As seen from the results, the RMS values were slightly higher than the orbit overlap results for the radial component.

**Figure 6:**
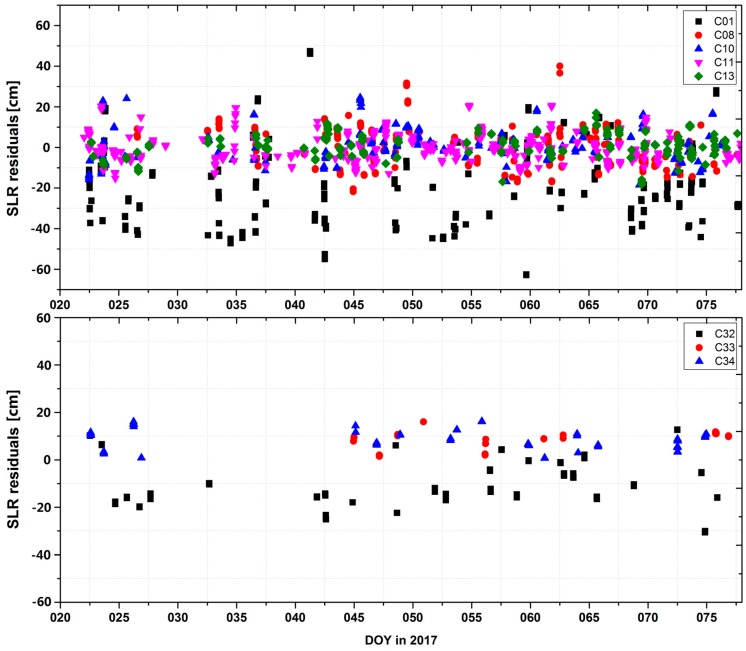
SLR residuals for BeiDou-2 satellites (top) and BeiDou-3e satellites (bottom).

As listed in Table 7, the GEO satellite C01 exhibited the worst performance, with a standard deviation (STD) of about 20 cm and a bias of about −19 cm. The STDs for all the IGSOs and MEOs among the tracked satellites were smaller than 10 cm, and there was no obvious bias for C08, C10, C11, and C13. With respect to the BeiDou-3e satellites, number of the tracking points was significantly fewer than those for the BeiDou-2 satellites. For BeiDou-3e satellites, C32 exhibited the worst performance, with an STD of about 10 cm and an obvious bias of approximately −10 cm in this calculation, which may be caused by inaccuracies in the PCO or LRA offset supplied by the OCC. C33 and C34 displayed a bias of about 8 cm, and their STDs were both about 4 cm, as shown in Figure 6.

### 4.3. Satellite Clock Offset Validation

**Figure 7:**
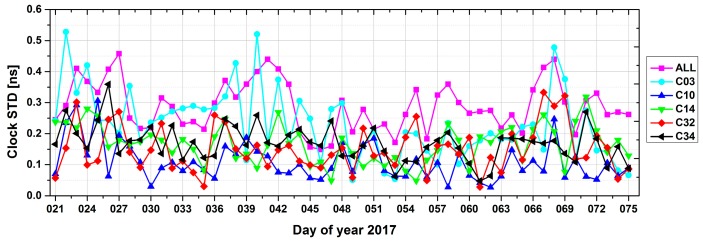
STD values for BeiDou clock products using one-day overlaps between two adjacent arcs with the middle day in the first arc as a reference.

In this study, satellite clock offsets were estimated from the undifferenced ionosphere-free code and phase combination of the B1I and B3I signals, together with orbit parameters. Because there was no external reference for BeiDou-3e satellites, differences in the clock offset series after removing reference clock data in overlapping arcs, in the same way as for the orbit overlaps, were used to assess the clock quality. Figure 7 shows the STDs for one-day clock offset overlaps between two adjacent arcs, where results for BeiDou-2 GEO (C03), BeiDou-2 IGSO (C10), BeiDou-2 MEO (C14), BeiDou-3e IGSO (C32), and BeiDou-3e MEO (C34) are illustrated, together with the mean values for all BeiDou-2 and BeiDou-3e satellites.

Note that, for MEOs, there is a substantial reduction in clock offsets due to the data gap outside the Asia-Pacific region. Thus, the statistics in Figure 7 and Table 8 only include clock offset within Asia-Pacific region for MEOs. As seen in Figure 7, MEOs displayed the best performance among all three types of satellites, with an average STD of 0.16 ns, followed by IGSOs (0.17 ns) and GEOs (0.22 ns), as listed in Table 8. As our analytical results show, the precision of the satellite clock offsets is consistent with that of the radial component of the orbit listed in Table 5, which is in line with theoretical expectations.

## 5. Precise Point Positioning (PPP)

To further analyze the improvements from BeiDou-2 to BeiDou-3e in PPP precision and convergence time, ambiguity fixed solutions for precise BeiDou orbits and clock offsets were utilized, together with IGS GPS products, and we set up five experiments. The five solutions are described and listed in Table 9. The first case uses only GPS observations, which can be used as baseline solution for comparisons. The second and third cases, namely GB114 and GB134, use combined GPS and BeiDou data but with different BeiDou constellations, that is, BeiDou-2 (PRN 1-14) and BeiDou-2/3e (PRN 1-14, 31-34) constellations, respectively, which can demonstrate the improvements by adding BeiDou observations. The last two cases represent the BeiDou only PPP performances, using BeiDou-2 only (PRN 1-14) and BeiDou-2/3e combined (PRN 1-14, 31-34) observations, respectively.

### 5.1. Static PPP Solutions

**Figure 8:**
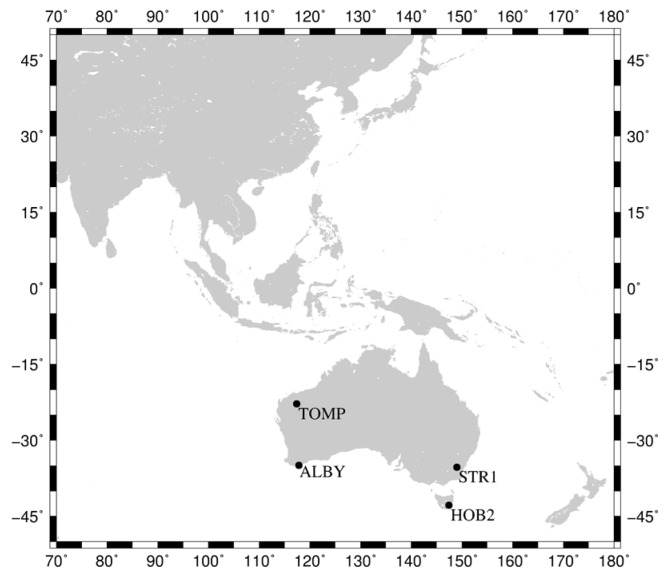
Stations used for static and kinematic PPP. Black points represent four stations, TOMP, ALBY, HOB2, and STR1, which are located in Australia.

For static PPP, considering data integrity and quality, four stations located in Australia were selected, namely, TOMP, ALBY, HOB2, and STR1. Their 24-hour solutions from DOY 052 to DOY 068 were calculated. We have adopted the following strategy, that is: when TOMP is used for PPP, the remaining stations are used for orbit and clock calculation without TOMP, the same as ALBY, HOB2 and STR1. Figure 8 shows the stations used for static and kinematic PPP.

**Figure 9:**
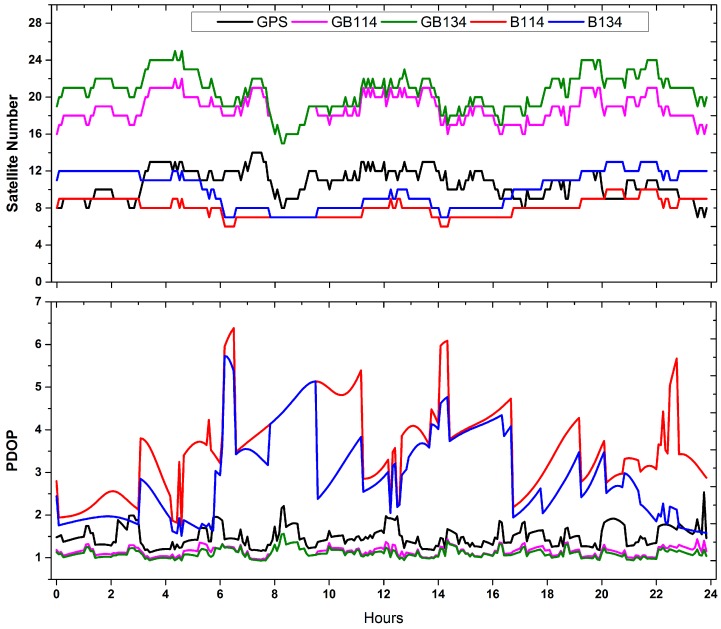
Variations in number of satellites used and position dilution of precision (PDOP) values of GPS, GB114, GB134, B114 and B134 solutions for station STR1 on DOY 057, 2017.

Figure 9 shows the satellite number and variations in position dilution of precision (PDOP) of GPS, GB114, GB134, B114 and B134 solutions for station STR1 on DOY 057, 2017. About eight BeiDou-2 satellites were tracked and the PDOP values ranged from 2 to 6, which presents the worst performance of space geometry in the selecting four cases. As depicted by Figure 9, GPS involved solutions (GPS/GB114/GB134) showed more stable overall tendency and better PDOP than BeiDou solutions (B114/B134). However, when one or more BeiDou-3e satellites were visible from the station, the PDOP performance could be improved. On average, PDOP of B134 (3.6) could be decreased by nearly 20%, compared with that of B114 (2.9). In addition, when about 20 GPS and BeiDou satellites could be tracked, the variations in the PDOP values of GB114 and GB134, which fluctuated around 1, were much smoother and steadier than that for the GPS-only solution.

We assessed the positioning precision using RMS values of the East (E), North (N), and Up (U) components. When compared with the corresponding ground truth values, the average coordinates calculated by IGS final orbit and clock products were used. The results are shown in Table 10.

**Figure 10:**
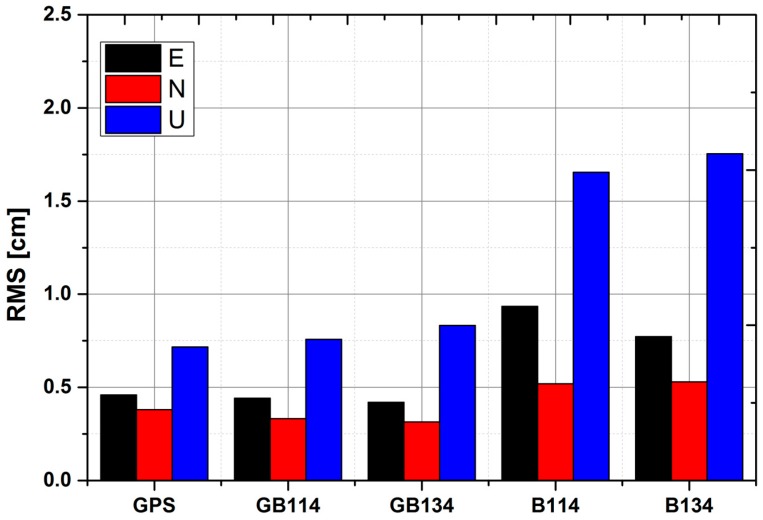
RMS values of precision for PPP in E, N, and U components at four stations (TOMP, ALBY, HOB2 and STR1) using five solutions compared with ground truth values.

Figure 10 shows the average RMS values for the four stations. The N components provided the best performance for all solutions, followed by the E and U components. The precision of solutions GPS, GB114 and GB134 mostly remained at the same level, with values of about 0.5 cm for the horizontal components and 0.8 cm for the vertical components. As depicted by Figure 10, for precisions in U components, GB134 performed little worse than GB114. The reasons maybe include as follows: PCO and PCV for BeiDou-2 series are corrected parameters supplied by Wuhan University [7], while PCO for BeiDou-3e satellites adopted in our analysis is initial values provided by OCC without PCV, as mentioned in Table 4 and Section 4.1, which may contain errors. Furthermore, precisions in U components are greatly influenced by corrected precision of Z direction in PCO. It is noteworthy that corrected value in Z direction for PCO is much larger than corrected values in X and Y, which results in relatively worse vertical precision for PPP. For the BeiDou-only solutions, a precision of 1.0 cm for the horizontal components and 1.7 cm for the vertical component were achieved. For the four solutions GB114, GB134, B134, and B114, it is noteworthy that when BeiDou-3e satellites were added, the performance for the horizontal components was improved to a limited extent. As shown in Figure 10, the horizontal and vertical precisions of BeiDou-only solutions are both lower than those of solutions GPS, GB114 and GB134. This may be caused by the relatively bad geometry of BeiDou, which can be presented by PDOP, as shown in Figure 9.

### 5.2. Kinematic PPP Solutions

**Figure 11:**
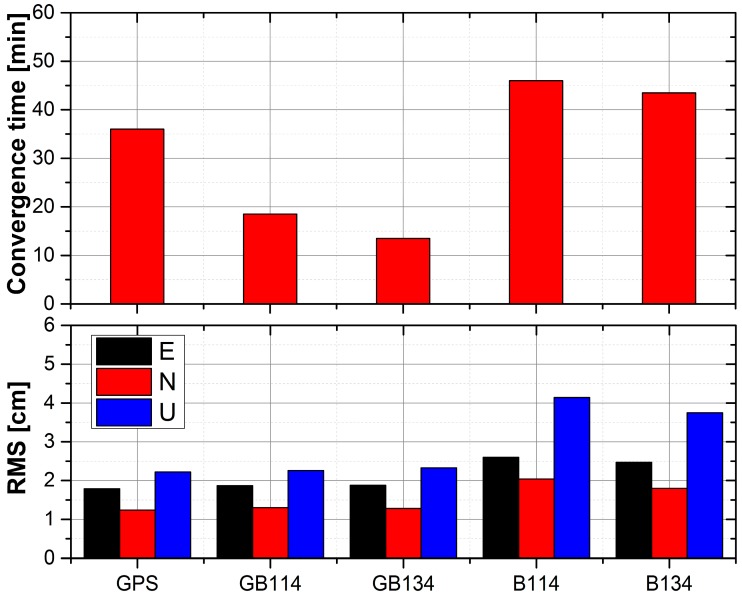
Average convergence times and RMS values for kinematic PPP at the TOMP, ALBY, HOB2 and STR1. The top panel shows the convergence times using five solutions, and the RMS values of the E, N, and U components are illustrated in the bottom panel.

For the kinematic PPP solutions, we calculated the convergence times for four stations, namely, TOMP, ALBY, HOB2 and STR1, during the period from DOY 052 to DOY 068. RMS values of the E, N, and U components for these four stations after convergence were also obtained. The data were recorded in 30-second intervals. Considering the positioning errors caused by orbit and clock interpolation, the first and last 3 h data were abandoned in this study. The condition for convergence in kinematic PPP is that if the three-dimensional RMS values for the current epoch and the following ten epochs are all less than 0.1 m, the current epoch is set as the convergence time [45]. It should be noted that the statistical results listed here are the RMS values of the E, N, and U components after convergence. Table 11 lists the precisions and convergence times obtained for the four stations using different kinematic PPP solutions. Figure 11 shows the RMS values of the E, N, and U components and convergence times for the four stations using different solutions. 

Among all the solutions, in terms of the RMS values, kinematic PPP using the GPS-only solution performed best for both horizontal and vertical components. Moreover, the results of kinematic PPP using GPS, GB114 and GB134 solutions were comparable and were all superior to those obtained using B134 and B114, where B114 performed worst. For convergence time, kinematic PPP using GPS-only solution needed 36 min, but when BeiDou solutions were included (GB114 and GB134), the convergence time for GB114 was improved significantly to 18.5 min, which was comparable to that reported by Li et al. [9]. Furthermore, when four BeiDou-3e satellites were involved, the convergence time was reduced to 13.5 min.

In the case of kinematic PPP, the RMS values were much higher than those for static solutions. This may because the position coordinates are estimated in epoch wise, where a random walk model was utilized between epochs. When we compared the B134 and B114 solutions, the inclusion of BeiDou-3e satellites could improve the geometric distributions of satellites and shorten the convergence time greatly.

## 6. Conclusions

The plan for global coverage by BeiDou was launched in 2015, and there are currently five next-generation satellites, named as BeiDou-3e, in orbit for validation and testing. Several iGMAS and GA stations were employed to analyze the characteristic performance of the original B1I and B3I signals via MP combinations in comparison with that of BeiDou-2 satellites in this study. As shown by our results, there was no obvious satellite-induced code bias for the next-generation BeiDou satellites, in contrast to the BeiDou-2 satellites. Using the real-valued ambiguity solution, the three-dimensional orbit consistency of BeiDou-2 GEOs, IGSOs, and MEOs reached 62 cm, 24 cm, and 23 cm, respectively, and the consistency of the radial component achieved 6 cm, 5 cm, and 5 cm, respectively. In comparison with BeiDou-2 satellites, the three-dimensional orbit consistency of BeiDou-3e IGSOs and MEOs reached 27 cm and 25 cm, respectively, and the consistency of the radial component achieved 7 cm and 5 cm, respectively. The ambiguity fixed solution was improved by about 10% in the along-track and cross-track components for IGSOs, but for MEOs the improvement was not noticeable. The results of SLR validation showed that, for BeiDou-2 satellites, the STD values for IGSOs ranged from 6.2 cm to 9.8 cm and STD for MEOs were about 6.8 cm, whereas for BeiDou-3e satellites, the STD values were 4 cm for MEOs and 9.6 cm for IGSOs. Furthermore, the clock quality was evaluated for BeiDou satellites, which indicated that the performance of clock products was in accordance with the orbit precision results.

PPP performances were analyzed with different GPS/BeiDou satellite configurations. Static PPP results show that including BeiDou-3e satellites can slightly improve the horizontal precision for BeiDou only or GPS/BeiDou combined solutions. The post-mission kinematic PPP convergence time using only BeiDou-2 satellites was the longest at about 46 min, but when GPS satellites were included, the convergence time was greatly reduced to about 18.5 min. As our analytical results showed, the newly launched BeiDou-3e satellites could improve the positioning precision and convergence time owing to their better geometry. The convergence time for kinematic PPP when combing GPS, BeiDou-2 and BeiDou-3e data was reduced to 13.5 min.

The results obtained in this study show that BeiDou-3e satellites can now achieve similar orbit and clock offset precision as BeiDou-2 satellites. By in-cooperating BeiDou-2 with BeiDou-3e satellites, the static and kinematic PPP performances are slightly improved. It is expected the BeiDou system can benefit the community much more after the whole global constellation is deployed.

## Figures and Tables

**Table 1:** Information on BeiDou-2- and BeiDou-3e- capable receivers. International GNSS Monitoring and Assessment Service (iGMAS).

Site	Location	Agency	Receiver Type	Antenna Type
ABJA	Nigeria, Abuja	iGMAS	gnss_ggr	RINT-8CH CETD
GUA1	China, Urumchi	iGMAS	gnss_ggr	RINT-8CH CETD
HMNS	South Africa, Hermanus	iGMAS	gnss_ggr	RINT-8CH CETD
BJF1	China, Beijing	iGMAS	CETC-54-GMR-4016	LEIAR25.R4 LEIT
BRCH	Germany, Braunschweig	iGMAS	CETC-54-GMR-4011	NOV750.R4 NOVS
DWIN	Australia, Darwin	iGMAS	CETC-54-GMR-4011	GNSS-750 NONE
KNDY	Sri Lanka, Kandy	iGMAS	CETC-54-GMR-4016	GNSS-750 NONE
LHA1	China, Lhasa	iGMAS	CETC-54-GMR-4016	NOV750.R4 NOVS
WUH1	China, Wuhan	iGMAS	CETC-54-GMR-4016	LEIAR25.R4 LEIT
ZHON	Antarctica, Zhongshan Station	iGMAS	CETC-54-GMR-4011	GNSS-750 NONE
ALBY	Australia, Albany	GA	SEPT POLARX5	JAVRINGANT_DM SCIS
ARUB	Australia, Arubiddy	GA	SEPT POLARX5	LEIAR25.R3 NONE
CEDU	Australia, Ceduna	GA	SEPT POLARX5	AOAD/M_T NONE
DAV1	Antarctica, Davis	GA	SEPT POLARX5	LEIAR25.R3 LEIT
HOB2	Australia, Hobart	GA	SEPT POLARX5	AOAD/M_T NONE
KUNU	Australia, Kununurra	GA	SEPT POLARX5	JAVRINGANT_DM SCIS
MEDO	Australia, Meadow Station	GA	SEPT POLARX5	LEIAR25.R3 LEIT
NCLF	Australia, Northcliffe	GA	SEPT POLARX5	JAVRINGANT_DM SCIS
NORS	Australia, Norseman	GA	SEPT POLARX5	JAVRINGANT_DM SCIS
PTHL	Australia, Port Hedland	GA	SEPT POLARX5	LEIAR25.R3 LEIT
STR1	Australia, Canberra	GA	SEPT POLARX5	ASH701945C_M NONE
THEV	Australia, Thevenard	GA	SEPT POLARX5	LEIAR25.R3 LEIT
TOMP	Australia, Tom Price	GA	SEPT POLARX5	LEIAR25.R3 LEIT
WILU	Australia, Wiluna	GA	SEPT POLARX5	LEIAR25.R3 LEIT

**Table 2:** Mean and root mean square (RMS) values (cm) of MP combinations for BeiDou-2, BeiDou-3e, and GPS satellites.

	BeiDou-2 IGSO		BeiDou-2 MEO		BeiDou-3e IGSO		BeiDou-3e MEO		GPS	
	B1	B3	B1	B3	B1	B3	B1	B3	L1	L2
**Mean**	1.8	1.5	1.2	1.1	0.2	0.1	−0.3	0.3	−0.1	−0.1
**RMS**	40.7	23.8	52.5	30.3	33.1	21.9	29.9	26.0	34.1	34.3

**Table 3:** Observational models and force models used in POD processing strategy. Phase center offset (PCO); phase center variation (PCV); zenith troposphere delays (ZTDs); Empirical CODE Orbit Model (ECOM).

Item	Model	Reference
Basic observations	Undifferenced ionosphere-free code and phase combination of B1I and B3I signals	
Sampling interval	300 s	
Cutoff elevation	10°	
Arc length	3 days	
Weighting	Prior precision of phase and code observations is 2 cm and 2 m, respectively, and elevation-dependent data weighting	
Satellite antenna phase center	C06-C14: PCO and PCV corrected with values estimated by Wuhan University (except C13); C31-C34: corrected with data supplied by BeiDou Operational Control Center (OCC)	[7]
Tropospheric delay	Saastamoinen model, global mapping function, two-hourly ZTDs	[37,38]
Relativity effect	Considered according to IERS Conventions 2010	[39]
Earth orientation parameters	Fixed according to IERS C04	[40]
Geopotential	EIGEN_GL04C up to degree 12 × 12 (European Improved Gravity model of the Earth by New techniques)	
N-body gravitation	Sun, Moon, and other planets: JPL DE405 (Jet Propulsion Laboratory Development Ephemeris 405) ephemeris used	
Solar radiation	ECOM five-parameter model with a constant acceleration bias in along-track direction	[28]
Attitude model	Both YS and ON models for BeiDou-2; YS model only for BeiDou-3e	

**Table 4:** PCO values for BeiDou-3e satellites (cm).

	PCO		
	X	Y	Z
**C31**	−5	0	100
**C32**	−4.5	−30	250
**C33**	−20	0	150
**C34**	−20	0	150

**Table 5:** Results of overlap comparison of real-valued ambiguity solution for BeiDou-2 and BeiDou-3e satellites in along-track, cross-track, radial and three-dimensional (3D) directions (cm). Pseudo random noise (PRN).

Satellite Type	PRN	Along-Track	Cross-Track	Radial	3D
BeiDou-2 GEO	C01	46.6	4.3	5.5	47.1
	C02	51.8	4.7	6.0	52.4
	C03	61.1	3.2	5.7	61.4
	C04	71.7	6.7	5.8	72.2
	C05	73.4	8.0	7.6	74.2
BeiDou-2 IGSO	C06	13.4	19.4	5.1	24.1
	C07	14.0	13.2	4.8	19.9
	C08	14.8	15.5	4.8	21.9
	C09	15.5	18.7	5.4	24.9
	C10	14.8	13.3	4.4	20.4
	C13	14.9	16.9	4.8	23.0
BeiDou-2 MEO	C11	17.3	12.8	4.8	22.0
	C12	15.5	11.5	4.3	19.7
	C14	15.6	11.6	4.9	20.0
BeiDou-3e IGSO	C31	21.5	15.7	6.5	27.4
	C32	16.9	15.1	5.5	23.3
BeiDou-3e MEO	C33	17.1	12.3	4.8	21.6
	C34	20.2	15.1	5.5	25.8

**Table 6:** Satellite laser ranging (SLR) offsets for BeiDou-2 and BeiDou-3e satellites (cm).

	SLR Offsets		
	X	Y	Z
C01	−54.38	−57.04	109.30
C08	−40.03	−57.30	109.34
C10	−40.23	−57.30	110.00
C11	−37.54	−53.80	110.00
C13	−40.23	−57.30	110.00
C31	−95.91	18.13	63.76
C32	18.51	68.54	196.02
C33	61.21	−7.17	122.90
C34	61.01	−7.12	124.48

**Table 7:** SLR validation of BeiDou-2 and BeiDou-3e orbits. STD, MEAN, and RMS represent the standard deviation, mean value, and root mean square of the two-way SLR residual, NP# and NP(U) represent the number of tracking points and points from which the data were used, and % represents the data utilization percentage (units: cm vs. adimensional).

PRN	STD	MEAN	RMS	NP#	NP(U)	%
C01	20.0	−18.9	27.5	305	305	100
C08	9.8	−0.3	9.8	345	331	96
C10	8.3	0.8	8.4	270	256	95
C11	6.8	−0.6	6.8	499	484	97
C13	6.2	0.7	6.2	293	293	100
C32	9.6	−9.5	13.9	85	85	100
C33	3.8	8.1	8.9	33	33	100
C34	4.2	8.6	9.5	58	58	100

**Table 8:** Average STD values for BeiDou-2 and BeiDou-3e clock offset in overlapping arcs with the middle day in the first arc as a reference (units: ns).

	BeiDou-2 GEO	BeiDou-2 IGSO	BeiDou-2 MEO	BeiDou-3e IGSO	Beidou-3e MEO	ALL
STD	0.22	0.17	0.15	0.18	0.17	0.18

**Table 9:** GPS and BeiDou solutions utilized to achieve precise point positioning (PPP).

Solution	Description
GPS	GPS only
GB114	GPS and BeiDou PRN 1-14
GB134	GPS and BeiDou PRN 1-14, 31-34
B114	BeiDou PRN 1-14
B134	BeiDou PRN 1-14, 31-34

**Table 10:** Precision of GPS and BeiDou solutions for static PPP of E, N, and U components at four stations (TOMP, ALBY, HOB2, and STR1) calculated during the period from DOY 052 to DOY 068, 2017 (RMS values in cm). East (E); North (N); Up (U).

	Site	TOMP			ALBY			HOB2			STR1			Average		
Sol.		E	N	U	E	N	U	E	N	U	E	N	U	E	N	U
**GPS**		0.55	0.52	1.26	0.49	0.34	0.79	0.38	0.36	0.39	0.40	0.30	0.43	0.46	0.38	0.72
**GB114**		0.37	0.46	1.06	0.62	0.37	0.90	0.37	0.30	0.48	0.41	0.20	0.59	0.44	0.33	0.76
**GB134**		0.40	0.30	1.34	0.54	0.44	0.86	0.36	0.32	0.49	0.38	0.20	0.64	0.42	0.32	0.83
**B134**		0.73	0.34	1.90	0.75	0.68	1.47	0.77	0.58	1.86	0.84	0.52	1.79	0.77	0.53	1.76
**B114**		0.71	0.53	1.41	0.91	0.59	1.76	1.25	0.49	1.84	0.87	0.47	1.61	0.94	0.52	1.66

**Table 11:** RMS values of GPS, GB114, GB134, B134 and B114 solutions at the TOMP, ALBY, HOB2 and STR1 for kinematic PPP in the E, N, and U components (calculated above) and relative convergence times (Con. Time). The RMS values are in cm and the convergence times are in minutes.

	Site	TOMP			ALBY			HOB2			STR1			Average			
Sol.		E	N	U	E	N	U	E	N	U	E	N	U	E	N	U	Con. Time
GPS		1.81	1.26	2.23	1.81	1.25	2.23	1.77	1.22	2.20	1.79	1.23	2.23	1.79	1.24	2.22	36
GB114		1.84	1.28	2.24	1.90	1.33	2.29	1.88	1.32	2.26	1.87	1.29	2.24	1.87	1.30	2.26	18.5
GB134		1.91	1.27	2.32	1.88	1.29	2.34	1.86	1.25	2.32	1.88	1.32	2.33	1.88	1.28	2.33	13.5
B134		2.42	1.75	3.68	2.48	1.83	3.61	2.50	1.83	3.78	2.48	1.81	3.94	2.47	1.80	3.75	43.5
B114		2.56	2.00	3.93	2.59	2.03	3.97	2.64	2.07	4.20	2.62	2.05	4.45	2.60	2.04	4.14	46

## Abbreviations

Acronym	Full Name
ACs	Analysis Centers
AR	Ambiguity Resolution
CNES/CLS	Centre National d’Etudes Spatiales/Collecte de Localisation Satellite
CODE	Center for Orbit Determination in Europe
DOY	Day Of Year
ECOM	Empirical CODE Orbit Model
GA	Geoscience Australia
GEO	GEostationary Orbit
GFZ	GeoForschungsZentrum Potsdam
GLONASS	GLObal Navigation Satellite System
GPS	Global Positioning System
HMW	Hatch–Melbourne–Wübbena combination
IERS	International Earth Rotation Service
iGMAS	international GNSS Monitoring and Assessment Service
IGS	International GNSS Service
IGSO	Inclined GeoSynchronous Orbit
ILRS	International Laser Ranging Service
ISL	Inter-Satellite Link
JAXA	Japan Aerospace Exploration Agency
LRA	Laser Retroreflector Array
MEO	Medium Earth Orbit
MGEX	Multi-GNSS Experiment Extension
NL	Narrow-Lane
NPs	Normal Points
ON	Orbit-Normal
PANDA	Position And Navigation Data Analyst software
PCO	Phase Center Offset
PCV	Phase Center Variation
PDOP	Position Dilution Of Precision
POD	Precise Orbit Determination
PPP	Precise Point Positioning
PRN	Pseudo Random Noise
RMS	Root Mean Square
SLR	Satellite Laser Ranging
SRP	Solar Radiation Pressure
STD	STandard Deviation
TUM	Technische Universität München
WHU	Wuhan University
WL	Wide-Lane
YS	Yaw-Steering
ZTDs	Zenith Troposphere Delays
